# Evaluation of Respirable Crystalline Silica in High School Ceramics Classrooms

**DOI:** 10.3390/ijerph110201250

**Published:** 2014-01-23

**Authors:** Matthew Fechser, Victor Alaves, Rodney Larson, Darrah Sleeth

**Affiliations:** 1 Rocky Mountain Center for Occupational and Environmental Health, Department of Family and Preventive Medicine, University of Utah, Salt Lake City, UT 84108, USA; E-Mails: mfechser@gmail.com (M.F.); rod.larson@hsc.utah.edu (R.L.); 2 Division of Environmental Health, Salt Lake County Health Department, Murray, UT 84107, USA; E-Mail: valaves@slco.org

**Keywords:** crystalline silica, respirable dust, particulate matter, ceramics, pottery, teachers, schools

## Abstract

Air concentrations of respirable crystalline silica were measured in eleven (11) high school ceramics classrooms located in Salt Lake County, UT, USA. Respirable dust was collected on PVC filters using precision flow pumps and cyclone samplers (n = 44). Filters were subsequently analyzed for respirable dust and percent crystalline silica content. The geometric mean of the silica concentrations was 0.009 mg/m^3^ near the teacher’s work station and 0.008 mg/m^3^ near the kilns. The number of students in the classroom was correlated to the silica concentration in the ceramics classroom, but no correlation was found between the silica concentrations and either the size of the classroom or the age of the building. Results from this study indicate that ceramics teachers may be at an increased risk of exposure to crystalline silica based on the ACGIH TLV of 0.025 mg/m^3^, with an exceedance of 21%.

## 1. Introduction

The abundance of crystalline silica has made exposures to it, along with the health effects associated with exposure, common and well documented. Historically, the primary disease associated with exposure to silica is silicosis [1]. The risk of developing silicosis increases with increasing cumulative exposure [2,3]. More recent studies have suggested that silica may be partially soluble in lung fluid [4] and as such may lead to other diseases including: end-stage kidney disease, arthritis, and other autoimmune disorders [5,6]. In addition, exposures to crystalline silica have been shown to increase the risk of developing tuberculosis in those who already have the latent form [7]. Many studies have identified an association between exposure to crystalline silica and the development of lung cancer [8,9]. In 1997, The International Agency for Research on Cancer (IARC) reviewed the available epidemiological data on silica and identified respirable crystalline silica as a human carcinogen [8,10].

The USA Occupational Safety and Health Administration (OSHA) has established a Permissible Exposure Limit (PEL) for respirable crystalline silica based on percent silica (% SiO_2_) content in aerosols as calculated by the following equation:

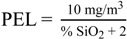
(1)


The USA National Institute for Occupational Safety and Health (NIOSH) indicates that OSHA’s current PEL does not adequately protect workers from the health effects associated with crystalline silica [6]. The American Conference of Governmental Industrial Hygienists (ACGIH) have established a more protective occupational exposure limit of 0.025 mg/m^3^ for quartz and cristobalite [11].

Exposures to silica can occur in a variety of industries, including any industry which involves the movement of sand [6]. Workers in the pottery and ceramics industry have been identified as being at risk of crystalline silica exposure [3,12,13,14,15]. Many of the materials used in the pottery manufacturing process (e.g., silica flours) can contain high concentrations of respirable crystalline silica and thereby increase the risk of exposure when being handled. Along with the handling and mixing of materials containing silica, the making of ceramics also uses kilns which can convert the quartz and non-crystalline silica into the crystalline forms: cristobalite and tridymite [6,10,16]. Epidemiology studies have also found that pottery workers have an increased risk of lung cancer, which could be related to silica exposure [17].

Many high schools provide ceramics classes for interested students and have ceramics classrooms dedicated to this activity. A potential source of silica exposure might therefore exist for high school ceramics teachers who handle such materials every day as part of their curriculum. This is especially true in poorly ventilated rooms, which may be present in older school buildings. Ventilation could also be adversely affected in winter, as windows and doors would be kept closed to keep the classrooms warmer. There are currently extremely limited data on exposure to silica in this setting [18] and the non-industrial nature of this environment may reduce concern for this potential exposure. The purpose of this study was therefore to assess the airborne concentration levels of respirable crystalline silica in high school classrooms during the winter. This information can be used to address the potential exposures that high school teachers might be encountering.

## 2. Methods

All 28 public high schools in Salt Lake County are subject to yearly health inspections conducted by the Salt Lake County Health Department (SLCHD); of these, 23 high schools offer a ceramics program. This inspection date is different each year and is unknown to the schools. Schools having a ceramics program that were due for inspection were selected for this study based on the next available inspection date. These inspections were not due to complaints and the schools were required to allow sampling to take place as part of this inspection. This resulted in 100% participation and eliminated the possibility of selection bias related to voluntary participation. Ultimately, eleven (11) schools with ceramics classrooms were randomly selected out of the 23 high schools in Salt Lake County with such curriculum. The characteristics of the 11 schools in this study are: (1) geographically scattered throughout the county, (2) pertain to four different school districts, and (3) encompass buildings built during different time periods (from the 1890s to the 2000s). Thus, this group of schools studied, representing nearly half of all applicable schools, provides a diverse selection of classroom sizes, ventilation systems, and maintenance methods.

Airborne concentrations of respirable crystalline silica were assessed by area sampling. Many teachers would split their time in the ceramics classrooms with other teachers, so by doing area sampling *versus* personal sampling it ensured that the sampling was all conducted in the ceramics classroom. Sampling was accomplished by following NIOSH method 7500 (Silica, Crystalline, by XRD) [19]. This method requires a 37 mm polystyrene cassette with a 5 µm pore size polyvinyl chloride (PVC) filter be attached to a cyclone centrifugal separator to collect a respirable fraction of airborne particulate. Most of the air samples (n = 38) were collected using GS-3 cyclone pre-separators (SKC, Inc., Eighty-four, PA, USA). However, in a few instances (identified in Table 1 and Table 2, see Results section), GS-1 (n = 2) or aluminum cyclones (n = 4) were used (SKC, Inc.). It should be noted that side-by-side comparison of these different cyclones could potentially provide different results, although the small number of samples taken with the other cyclones preclude such analysis. AirChek 52 pumps (SKC, Inc.) were used to pull air through the samplers. The flow rate for each pump was set as recommended by the manufacturer of the cyclone so that the respirable fraction of dust would be collected on the filter (4 µm cut-point). The pump flow rate for GS-1 cyclones was 2.0 liters/min; for GS-3 cyclones was 2.75 liters/min, and for aluminum cyclones the flow rate was 2.5 liters/min. The pump flow rates were calibrated using a Bios DryCal Defender (Mesa Laboratories Inc., Lakewood, CO, USA) primary flow standard calibrator. 

Each selected school had two samplers set up in the school’s ceramics classroom. One sampling device was placed in a location identified by the teacher as where he/she spent most of the time. This sampling device was typically placed near the teacher’s desk (located in different locations at the various schools) in each sampled ceramics classroom. Another sampling device was placed near the kiln in a room attached to the ceramics classroom. Two control samples were used, with one control sample being set up in a regular classroom or office where exposure to crystalline silica would not be expected. The other control sample was placed outside the school building away from any building exhaust vents. Outside sampling devices were placed within one meter of the school building, except when a more secure area was selected by the custodial staff. No sampling device was more than five meters from the school building. The sampling devices were operated for the entire work day of the teacher, approximately eight hours. For every ten samples collected, a field blank was created by opening a cassette with a new filter inside and then immediately closing the cassette. The field blanks were submitted along with the collected samples to identify the possibility of sample contamination. 

Sampling areas were marked off with signs to discourage students and teachers from tampering with the sampling devices. A photo was taken of each sampling device at the start of the sampling period and another was taken after sample collection had been completed for the day to ensure there was no tampering with the sample collection equipment. Teachers were instructed to keep windows and doors to the ceramics classroom closed. Additional data that were also collected for each ceramics classroom sampled included:

- Location and number of air vents in the classroom
- Start and stop time of each sampling pump
- Flow rate of sampling pump at the start and stop of the sample collection period
- Location of the kiln in room
- Any form of local exhaust ventilation in the classroom
- Age of the school
- Room size (in m^3^)
- Total number of students in attendance during the day of the sample collection

The teachers were instructed to ensure that the kilns would be operated on the day of sampling. Although specific information was not collected from each teacher, most materials were ordered from the same supplier. On inspection of the material safety data sheets (MSDS), silica was identified in many products that would typically be used. Additionally, it was determined that most material handling was carried out while wet, with no projects being made when the material was mostly dry.

The collected samples were hand delivered to ALS Environmental Laboratory for analysis. ALS is accredited by the American Industrial Hygiene Association (AIHA) for NIOSH method 7500. The Limit of Detection (LOD) for measuring the total respirable dust on the filters using NIOSH method 7500 was 20 µg and the LOD for respirable crystalline silica was 10 µg. Regression on order statistics were used to substitute values below the LOD for statistical analysis [20,21].

### Data Analysis Methods

The data were tested for normality and log-transformed for analysis. The geometric mean (GM), geometric standard deviation (GSD), median and the 25th and 75th percentiles were calculated. Regression analysis was conducted to look at the impact of the number of students, the size of the classroom, and the age of the school on the concentration of respirable dust and respirable silica in the ceramics classrooms, both for univariate and multiple regressions. ANOVA was used to determine if the respirable dust and silica concentrations were statistically significantly different between the classroom and the kiln. Pearson Correlation Coefficients were also calculated between the classroom and kiln measurements, for both the silica and respirable dust concentrations. Statistical analysis was performed using SAS 9.3 (SAS Institute, Cary, NC, USA). A *p* value of less than 0.05 was considered grounds for rejecting the null hypothesis.

The results were compared against the OSHA Permissible Exposure Limit (PEL) and against the American Conference of Governmental Industrial Hygienists (ACGIH) Threshold Limit Value (TLV) for crystalline silica. The probability of exceeding the TLV was calculated. Both the PEL and TLV are values based on personal sampling, *i.e.*, collecting the air sample in the individual’s breathing zone. Since the data collected were area-based, the TLV and PEL do not directly apply to the results, but can be useful in identifying potential risk areas [11,22].

## 3. Results

### 3.1. Respirable Dust

The GM and GSD of the respirable dust concentration in the ceramics classrooms were 0.054 mg/m^3^ and 4.24, respectively. The median was 0.055 mg/m^3^, with 25th percentile of 0.029 mg/m^3^ and 75th percentile of 0.101 mg/m^3^. The GM and GSD of the respirable dust concentration by the kilns were 0.029 mg/m^3^ and 3.39, respectively. The median concentration by the kilns was 0.033 mg/m^3^, with 25th percentile of 0.017 mg/m^3^ and 75th percentile of 0.050 mg/m^3^. Only one of the control samples in another classroom and outside the school building had detectable levels of respirable dust. These values were all well below the PEL and TLV for respirable dust (5 mg/m^3^ and 3 mg/m^3^, respectively). The respirable dust concentrations in the classroom were not significantly different from near the kiln (*p* value = 0.296, R^2^ = 0.06), but these concentrations were highly correlated (r = 0.81). The results for respirable dust are displayed in Table 1. All blank samples were below the limit of detection.

**Table 1:** Summary of results for respirable dust concentrations by location.

School No.	Respirable Dust Concentration (mg/m^3^)			
	Classroom	Kiln	Control #1 (Inside)	Control #2 (Outside)
1	0.077	0.034	<0.015	<0.015
2	<0.015	<0.015	<0.016	<0.016
3	<0.015	<0.015	<0.015	<0.015
4	0.120	0.031	<0.015	<0.015
5	0.016	0.033	<0.015	<0.015
6	0.055	<0.015	<0.015	0.018
7	0.360	0.280	<0.014	<0.017 ^a^
8	0.530	0.069	<0.015	<0.017 ^a^
9	0.040 ^a^	<0.015 ^b^	<0.022 ^b^	N/A ^a^
10	0.091	0.081	0.021	<0.015
11	0.050	0.058	<0.016	<0.015
Median (25%–75%)	0.055 (0.029–0.101)	0.033 (0.017–0.050)	N/A	N/A
Geometric Mean(GSD)	0.054 (4.24)	0.029 (3.39)	N/A	N/A

Notes: ^**a**^ Aluminum cyclone; **^b^** GS-1 cyclone.

### 3.2. Respirable Crystalline Silica

The GM and GSD of the respirable crystalline silica concentrations in the ceramics classrooms were 0.009 mg/m^3^ and 3.75 respectively. The median concentration was 0.011 mg/m^3^, with 25th percentile of 0.005 mg/m^3^ and 75th percentile of 0.015 mg/m^3^. The GM and GSD of the silica concentrations by the kilns were 0.006 mg/m^3^ and 2.92, respectively. The median concentration by the kilns was 0.005 mg/m^3^, with 25th percentile of 0.004 mg/m^3^ and a 75th percentile of 0.009 mg/m^3^. Only one of the control concentrations was above the LOD. The percent silica in the samples that had measureable quantities had a range from 10% to 88%. The respirable silica concentrations in the classroom were not significantly different from near the kiln (*p* value = 0.401, R^2^ = 0.04), but these concentrations were highly correlated (r = 0.87). The results for respirable crystalline silica concentrations are displayed in Table 2. Again, all blank samples were below the limit of detection.

Comparing the respirable silica concentrations in the classroom and by the kiln against the ACGIH TLV, two classrooms and one kiln had silica concentrations above the TLV of 0.025 mg/m^3^. Based on industrial hygiene guidelines for exposure assessment [23,24] using the GM and GSD, the TLV exceedance in the classroom was 21% (95% CI: 9%, 42%). 

**Table 2:** Summary of results for respirable crystalline silica concentrations by location.

School No.	Respirable Crystalline Silica Concentration (mg/m^3^) (% Quartz)			
	Classroom	Kiln	Control #1 (Inside)	Control #2 (Outside)
1	0.015 (20%)	0.011 (33%)	<0.008	<0.008
2	<0.008	<0.008	<0.008	<0.008
3	<0.008	<0.008	<0.008	<0.008
4	0.022 (18%)	0.016 (51%)	<0.008	<0.008
5	0.014 (88%)	<0.008	<0.008	<0.008
6	<0.008	<0.008	<0.008	0.012 (65%)
7	0.043 (12%)	0.039 (14%)	<0.008	<0.008 ^a^
8	0.074 (14%)	0.012 (18%)	<0.008	<0.008 ^a^
9	<0.008 ^a^	<0.011 ^b^	<0.011 ^b^	N/A ^a^
10	0.011 (12%)	0.008 (10%)	<0.012 (55%)	<0.008
11	<0.008	<0.008	<0.008	<0.008
Median (25%–75%)	0.011 (0.005–0.015)	0.005 (0.004–0.009)	N/A	N/A
Geometric Mean (GSD)	0.009 (3.75)	0.006 (2.92)	N/A	N/A

Notes: ^**a**^ Aluminum cyclone; **^b^** GS-1 cyclone.

### 3.3. Regression Analysis

Table 3 shows the various school characteristics, including ceramics classroom volume, number of students in the classroom and the age of the school building. Univariate and multiple regression analysis, utilizing the log-transformed data, were used to identify any correlation between these variables and the concentrations found in the ceramics classroom and by the kiln. Ceramics classroom volume (total m^3^ for each classroom) was not a significant predictor of respirable dust concentration (*p* value = 0.645, R^2^ = 0.02) or crystalline silica concentration (*p* value = 0.352, R^2^ = 0.09) in the ceramics classroom. The number of students in the classroom was also not a significant predictor of respirable dust concentration (*p* value = 0.230, R^2^ = 0.16) but it was a significant predictor of respirable silica concentrations (*p* value = 0.028, R^2^ = 0.43) in the ceramics classroom. From this regression model it is estimated that increasing the number of students from 80 to 120 would increase the silica concentration from below the LOD to 0.022 mg/m^3^, which is approaching the TLV. The age of the school was not a significant predictor of the respirable dust concentration (*p* value = 0.801, R^2^ = 0.01) or respirable silica concentrations (*p* value = 0.618, R^2^ = 0.03). Multiple regression including all three variables was not significant for respirable dust in the classroom (*p* value = 0.699, R^2^ = 0.17) or silica in the classroom (*p* value = 0.198, R^2^ = 0.47). 

**Table 3:** School characteristics used for regression analysis.

School No.	Room Volume (m^3^)	Students No.	Age of Building (years)
1	453	116	3
2	363	65	14
3	569	93	32
4	269	100	18
5	483	129	47
6	413	98	32
7	389	117	23
8	720	108	28
9	272	55	113
10	351	88	51
11	217	79	34

## 4. Discussion

The respirable dust concentrations in each ceramics classroom were all below both the OSHA PEL (5 mg/m^3^) and the ACGIH TLV (3 mg/m^3^). This suggests that the respirable dust concentration levels were well within acceptable levels. The respirable crystalline silica concentrations were all below the PEL (as calculated from Equation 1), but some schools (two out of 11 in the ceramics classroom and one out of 11 by the kiln) were above the TLV (0.025 mg/m^3^) for an 8 h work day. From this, it is estimated that for every 100 days of working in a ceramics classroom (40 h per week), 21 would involve exposure over the current TLV, with an upper confidence limit of 42 days. These results indicate that teachers may be at an increased risk of adverse health effects caused by exposure to respirable silica. Additionally, concentrations by the kiln were found to be lower than those found in the classroom, although this difference was not statistically significantly different, and these measurements were highly correlated. This is likely due to the use of local exhausts ventilation in the design of the kilns.

It should be kept in mind that the collected data are for area sampling, and therefore the concentrations found in classrooms that exceeded ACGIH recommended exposure limits do not directly translate to high exposures for teachers. It does, however, indicate the potential for an increased risk of silica exposures above the ACGIH TLV. Personal sampling may reveal higher exposures than those obtained with area sampling because teachers are often closer to the source of the exposure.

Based on these statistical results, it was also determined that the number of students in attendance could not be used as a predictor of airborne respirable dust. Classrooms may have had a larger attendance over the day, but the average number of students in the classroom may not have varied as much between schools. Another possibility is that, due to budget constraints, larger classes were not able to create as many pieces as the smaller classes, so the actual amount of ceramics being produced were similar. Unfortunately, information regarding the number of ceramic pieces created or worked with in each classroom was not available to test that hypothesis. However, the number of students was correlated to the respirable silica concentrations. One explanation for this might be that although the materials were believed to be similar across all schools, perhaps those schools with more students were buying different (e.g., cheaper) material with higher silica content. 

The size of the classroom was also unable to be used as a predictor of airborne respirable dust or respirable silica concentrations. This could be due to differences in ventilation systems between large and small classrooms, although these systems were not tested as part of this study. The age of the schools was also unable to be used as a predictor of airborne respirable dust or respirable silica concentrations. It is likely that the age of the school does not necessarily affect the actual ventilation rates inside the classrooms. The relatively small sample size, including many concentrations under the limit of detection, may also limit the capabilities of this regression analysis to identify such predictors.

Study strengths included a randomization of school selection, with 100% participation by those schools selected. Additionally, schools were selected from across Salt Lake County, including several different school districts, making it more accurate to generalize results from this study to other geographic areas in other states. Air samples were collected for the full 8 h in the ceramics classroom, suggesting that measured concentrations are similar to what teachers might experience working the full day in their classrooms. 

An identified weakness in this study is that the ventilation rates in the classrooms were not evaluated. Additional future studies should address the ventilation rates and how they contribute to the airborne concentrations of crystalline silica. Another weakness of this study is the use of area samples instead of personal samples. Area samples cannot be directly compared against a PEL and TLV. Another weakness is that air sampling devices were unable to be observed throughout the entire collection day. However, to minimize this potential for sample tampering, photos were taken of the equipment pre- and post- sampling to assist in identifying if tampering may have occurred.

In addition to exposure to high school ceramics teachers, these results imply the possibility of short-term exposure potential for high school students in these classrooms. Although students do not spend a full eight hour day in this environment, presumably there may be higher exposures during the time of ceramics handling that poses a risk to them as well. Peak exposures—to both teachers and students—may therefore be an informative area of future study. 

## 5. Conclusions

The ceramics classrooms sampled in this study not only had increased levels of airborne respirable crystalline silica compared to other classrooms, but the levels were potentially above recommended occupational exposure limits. Teachers working in these classes therefore have an increased risk of being exposed to silica concentrations that may cause adverse health effects. Individual schools should evaluate the controls they have in place in their ceramics classrooms. If the possibility for high exposures is present, additional controls should be added to the ceramics classroom to reduce the amount of airborne particulate present. If necessary, personal sampling should be performed to determine the teacher’s exposure. Once additional controls have been put in place, ongoing industrial hygiene monitoring should be performed to ensure teacher exposures have been reduced.
